# P-800. Pharmacist-driven Methicillin-resistant Staphylococcus aureus Nasal Polymerase Chain Reaction (PCR) Screening in Multi-hospital System Reduced Resistant Gram-positive Antibiotics Use and Standardized Antimicrobial Administration Ratio (SAAR)

**DOI:** 10.1093/ofid/ofaf695.1010

**Published:** 2026-01-11

**Authors:** Chiraag Gupta, Holly Murphy, Jennifer Chou, James Shen, Curtis D Collins

**Affiliations:** Trinity Health Ann Arbor, Ypsilanti, MI; Trinity Health Ann Arbor, Ypsilanti, MI; Trinity Health Livonia, Livonia, Michigan; Trinity Health Oakland, Pontiac, Michigan; Trinity Health Ann Arbor, Ypsilanti, MI

## Abstract

**Background:**

Methicillin-resistant Staphylococcus aureus (MRSA) nasal polymerase chain reaction (PCR) testing, with known high specificity and negative predictive value, is particularly effective in ruling out MRSA pneumonia. There is significant potential to enhance antimicrobial stewardship efforts and optimize use of antibiotics for resistant gram-positive infections, especially when implemented and monitored in an evidence-based, comprehensive manner. In early 2023, health-system hospitals in Southeast Michigan authorized pharmacists to order MRSA nasal PCR swabs per protocol. This study aimed to investigate the impact of MRSA nasal PCR introduction combined with pharmacist active monitoring and intervention on the utilization of antibiotics for resistant gram-positive infections.

**Figure 1.**
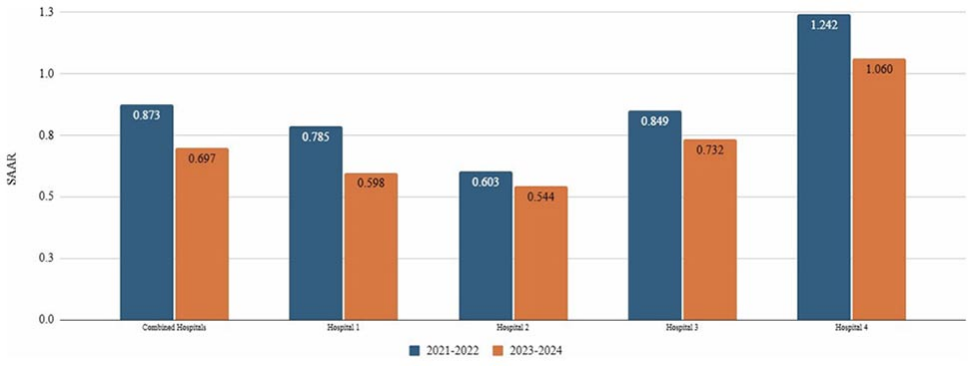
Resistant Gram-positive Agent Standardized Antimicrobial Administration Ratios Bar graph comparing Standardized Antimicrobial Administration Ratios (SAARs) for resistant gram-positive agents pre- and post-initiation of pharmacy-driven MRSA PCR nasal screening

**Methods:**

This multi-center, retrospective pre-post, cohort study evaluated utilization of antibiotics for resistant gram-positive infections in a multi-hospital health-system in Michigan. Cohorts were compared prior to (January 2021 to December 2022) and following MRSA PCR testing rollout (January 2023 to December 2024). Changes in resistant gram-positive agent days of therapy (DOTs) per 1,000 days present and Standardized Antimicrobial Administration Ratio (SAAR) data were evaluated between cohorts at the facility and patient care unit levels.

**Results:**

There was a significant decrease in resistant gram-positive agent utilization between cohorts demonstrated by a 20.2% decrease in SAAR (0.893 vs. 0.697; *P* < 0.001) (Figure 1). This resulted in a reduction of 8,124 DOTs over predicted historical use. Similarly, DOTs/1,000 days present decreased (88 DOTs/1000 days present vs. 63 DOTs/1000 days present; *P* < 0.001). All hospitals showed significant SAAR reductions and seventeen of 23 patient care units across all hospitals demonstrated significant SAAR decreases. This included a 31.2% decrease in SAARs in the intensive care units (0.988 vs. 0.68; *P* < 0.001*).*

**Conclusion:**

MRSA nasal PCR introduction combined with pharmacist active monitoring and intervention significantly improved utilization of resistant gram-positive agents across a multi-hospital health-system.

**Disclosures:**

All Authors: No reported disclosures

